# Diagnostic accuracy of first‐trimester combined screening for early‐onset and preterm pre‐eclampsia at 8–10 compared with 11–13 weeks' gestation

**DOI:** 10.1002/uog.22071

**Published:** 2021-01-02

**Authors:** M. Mendoza, P. Garcia‐Manau, S. Arévalo, M. Avilés, B. Serrano, M. á. Sánchez‐Durán, I. Garcia‐Ruiz, E. Bonacina, E. Carreras

**Affiliations:** ^1^ Maternal Fetal Medicine Unit, Department of Obstetrics Hospital Universitari Vall d'Hebron, Universitat Autònoma de Barcelona Barcelona Spain

**Keywords:** early‐onset pre‐eclampsia, first‐trimester, PlGF, pre‐eclampsia, screening, uterine artery Doppler

## Abstract

**Objectives:**

To compare the ability of first‐trimester combined screening for pre‐eclampsia (PE) to predict early‐onset and preterm PE when pregnancy‐associated plasma protein‐A (PAPP‐A) and placental growth factor (PlGF) were assessed before *vs* after 11 weeks' gestation.

**Methods:**

This was a secondary analysis of a prospective cohort study of singleton pregnancies undergoing routine first‐trimester screening conducted at Vall d'Hebron University Hospital, Barcelona, Spain, between October 2015 and September 2017. Demographic characteristics, obstetric history, maternal history and biophysical markers (mean uterine artery pulsatility index and mean arterial blood pressure (MAP)) were recorded at the first‐trimester scan (at 11 + 0 to 13 + 6 weeks' gestation). Maternal serum concentrations of PAPP‐A and PlGF were assessed from the routine first‐trimester blood test (at 8 + 0 to 13 + 6 weeks). Women were classified into two groups depending on whether serum biomarkers were assessed at 8 + 0 to 10 + 6 weeks or at 11 + 0 to 13 + 6 weeks. Probability scores for early‐onset and preterm PE were calculated by using two different algorithms: the multivariate Gaussian‐distribution model and The Fetal Medicine Foundation (FMF) competing‐risks model. Receiver‐operating‐characteristics (ROC) curves were produced and detection rates at fixed 5% and 10% false‐positive rates were computed to compare the performance of these algorithms when PAPP‐A and PlGF were assessed before *vs* after 11 weeks.

**Results:**

Of the 2641 women included, serum biomarkers were assessed before 11 weeks in 1675 (63.4%) and at or after 11 weeks in 966 (36.6%). Of these, 90 (3.4%) women developed PE, including 11 (0.4%) cases of early‐onset PE and 30 (1.1%) of preterm PE. Five (45.5%) cases of early‐onset and 16 (53.3%) of preterm PE were identified in the group in which serum biomarkers were assessed at 8 + 0 to 10 + 6 weeks and six (54.5%) cases of early‐onset and 14 (46.7%) of preterm PE in the group in which serum biomarkers were assessed at 11 + 0 to 13 + 6 weeks. In the prediction of early‐onset and preterm PE using the Gaussian algorithm, no differences were observed between the areas under the ROC curves (AUCs) when PAPP‐A and PlGF were measured before or after 11 weeks. In the prediction of early‐onset and preterm PE using the FMF algorithm, no differences were observed between AUCs for any of the combinations used for risk calculation when the serum biomarkers were obtained before *vs* after 11 weeks, except for the combination of PAPP‐A and MAP, which showed a greater AUC for the prediction of early‐onset PE when PAPP‐A was measured at or after 11 weeks.

**Conclusions:**

The prediction of early‐onset and preterm PE is similar when serum biomarkers are measured before or after 11 weeks. This allows the use of a two‐step approach for PE risk assessment that permits immediate risk calculation at the time of the first‐trimester scan. © 2020 The Authors. *Ultrasound in Obstetrics & Gynecology* published by John Wiley & Sons Ltd on behalf of International Society of Ultrasound in Obstetrics and Gynecology.

**CONTRIBUTION**

**What are the novel findings of this work?**

The performance of combined first‐trimester screening in predicting early‐onset and preterm pre‐eclampsia (PE) is similar when serum levels of pregnancy‐associated plasma protein‐A and placental growth factor are measured before or after 11 weeks' gestation.

**What are the clinical implications of this work?**

Measurement of serum biomarkers for both PE and aneuploidy screening before the first‐trimester scan permits risk calculation for both screening tests at the time of the scan without compromising PE screening performance.

## INTRODUCTION

Pre‐eclampsia (PE) occurs in approximately 2–8% of pregnancies^1^. It is the primary cause of maternal admission to the intensive care unit and is responsible for approximately 15% of all pregnancy‐related deaths in developed countries^2^, ^3^. In recent years, several studies published by The Fetal Medicine Foundation (FMF) have shown that their algorithm constructed by a combination of maternal history and biochemical and biophysical markers in the first trimester of pregnancy can effectively predict early‐onset and preterm PE^4^, allowing commencement of acetylsalicylic acid (ASA) before 16 weeks' gestation, which has proved to significantly reduce the risk of developing PE^5^.

Recently, our group participated in the development of a new first‐trimester Gaussian model constructed using the same variables as in the FMF algorithm, which also has a good performance in the prediction of early‐onset PE^6^. In both studies^4^, ^6^, maternal characteristics and biophysical markers were assessed at the time of the first‐trimester ultrasound examination (between 11 + 0 and 13 + 6 weeks). However, serum biomarkers were assessed between 11 + 0 and 13 + 6 weeks in the FMF study while in our study they were assessed between 8 + 0 and 13 + 6 weeks, preferably before the ultrasound assessment, using a two‐step approach. The reason for assessing serum biomarkers early in the first trimester was that the blood samples were drawn at the time of routine first‐trimester aneuploidy screening, which is usually done before the first‐trimester scan since the performance of first‐trimester biochemical screening for trisomy 21 is best done at 9–10 weeks^7^, ^8^. Placental growth factor (PlGF) is the most valuable biomarker used in both algorithms. Even though PlGF has been shown to have excellent precision and reliability from 5 + 0 weeks onwards for discriminating between PE and normal pregnancies^9^, a recent study found that its predictive capacity for PE increases after 11 weeks^10^. Therefore, it is reasonable to believe that the precision of these algorithms may drop when PlGF is measured before 11 weeks; however, the performance of these algorithms in the early and late first trimester has not been compared. The aim of this study was to evaluate the performance of the FMF and the Gaussian algorithms in predicting early‐onset and preterm PE when pregnancy‐associated plasma protein‐A (PAPP‐A) and PlGF were assessed before, compared with after, 11 weeks.

## METHODS

This was a secondary analysis of data from the population that participated in the development of the first‐trimester Gaussian algorithm to predict early‐onset PE^6^. That prospective cohort study was conducted at Vall d'Hebron University Hospital, Barcelona, Spain, from October 2015 to September 2017. The local ethics committee (CEIC‐VHIR PR(AMI)265/2018) approved the study protocol. A total of 3777 unselected singleton pregnant women attending for their routine first‐trimester scan (from 11 + 0 to 13 + 6 weeks) were invited to participate, of whom 2946 agreed and provided written informed consent. Of these, 305 (10.4%) participants were excluded owing to missing outcome data (*n* = 86), major fetal defects or chromosomopathies (*n* = 13), miscarriage or fetal death before 24 weeks (*n* = 15) or insufficient remaining blood sample to measure PlGF (*n* = 191). None of the remaining 2641 participants had received ASA at any time during their pregnancy.

After gestational age (GA) had been confirmed by fetal crown–rump length measurement during the scan^11^, demographic characteristics, obstetric history, maternal history, biophysical markers and biochemical markers were recorded in an electronic database. In all participants, transabdominal mean uterine artery pulsatility index (UtA‐PI)^12^ and mean arterial blood pressure (MAP) were assessed during the first‐trimester scan at 11 + 0 to 13 + 6 weeks. Maternal blood samples were analyzed to establish the serum concentrations of PAPP‐A and PlGF. One sample was obtained from each woman during the routine first‐trimester blood test for aneuploidy screening, at 8 + 0 to 13 + 6 weeks. Maternal serum levels of PAPP‐A (mU/L) and PlGF (pg/mL) were determined by fully automated Elecsys assays for PAPP‐A and PlGF on an immunoassay platform (Cobas e analyzers; Roche Diagnostics, Rotkreuz, Switzerland). As PAPP‐A and PlGF values change with GA, they were transformed to multiples of the median to be used in risk assessment^6^.

In all patients, blood pressure (BP) was measured at 11 + 0 to 13 + 6 weeks by a nurse using an automatic BP measurement device (Microlife WatchBP Home; Microlife Corporation, Taipei, Taiwan); a single measurement was obtained in one arm (right or left) after a 5‐min rest while the woman was seated. MAP was calculated as: diastolic BP + (systolic BP – diastolic BP)/3.

PE was defined according to the guidelines of the International Society for the Study of Hypertension in Pregnancy as systolic BP ≥ 140 mmHg and/or diastolic BP ≥ 90 mmHg confirmed by repeat measurements over a few hours, developing after 20 weeks' gestation in a previously normotensive woman, accompanied by proteinuria of ≥ 300 mg per 24 h or a spot urine protein‐to‐creatinine ratio ≥ 0.3 mg/mg or dipstick urinalysis ≥ 1+ when a quantitative method was not available^13^. Early‐onset and preterm PE were defined as PE necessitating delivery before 34 weeks and before 37 weeks, respectively.

Women were classified into two groups according to whether the blood sample for biomarker assessment was drawn before or after 11 weeks' gestation. We then coded the variables required for the prediction formulae according to the description provided in the articles and calculated the probability score for early‐onset PE using two different algorithms: the multivariate Gaussian‐distribution model and the FMF competing‐risks model^4^, ^6^.

Receiver‐operating‐characteristics (ROC) curves were produced and detection rates at fixed 5% and 10% false‐positive rates (FPR) were computed for all combinations of markers involved in the risk assessment, to compare the performance of the two algorithms for predicting early‐onset and preterm PE when PAPP‐A and PlGF were measured before and after 11 weeks^14^.

### Statistical analysis

Rcmdr package for R version 2.3‐1 software (The R Foundation, Vienna, Austria) was used for statistical analysis. Differences in categorical data between the groups were assessed using the χ‐square or Fisher's exact test, as appropriate, and are reported as *n* (%). Differences in continuous variables between the groups were assessed using the Mann–Whitney *U*‐test and are reported as median (interquartile range). Statistical significance was set at *P* < 0.05.

## RESULTS

PAPP‐A and PlGF were assessed before 11 weeks in 1675 (63.4%) of the 2641 women, and at or after 11 weeks in 966 (36.6%). Ninety (3.4%) women developed PE, including 30 (1.1%) cases of preterm PE and 11 (0.4%) of early‐onset PE. Five (45.5%) cases of early‐onset and 16 (53.3%) of preterm PE were identified in the group in which serum biomarkers were assessed at 8 + 0 to 10 + 6 weeks and six (54.5%) cases of early‐onset and 14 (46.7%) of preterm PE in the group in which serum biomarkers were assessed at 11 + 0 to 13 + 6 weeks.

Baseline characteristics of the study population were compared between the two groups (assessment of biochemical markers before 11 *vs* at or after 11 weeks) and are shown in Table 1. In women who developed early‐onset PE, no significant differences were observed between the two groups apart from GA at the time of PAPP‐A and PlGF assessment. In women who developed preterm PE, PAPP‐A and PlGF levels and the GA at their measurement were significantly different between the two groups. When non‐affected women were evaluated, significant differences were observed between the two groups in ethnicity, smoking status, obstetric history, PAPP‐A and PlGF levels and GA at their measurement, UtA‐PI and GA at ultrasound assessment.

**Table 1:** Baseline characteristics and biophysical and biochemical measurements in women who developed early‐onset (before 34 weeks) pre‐eclampsia (PE), those who developed preterm (before 37 weeks) PE and those who did not develop PE before 37 weeks' gestation, according to whether pregnancy‐associated plasma protein‐A (PAPP‐A) and placental growth factor (PlGF) were measured before or after 11 weeks

Parameter	PE before 34 + 0 weeks (*n* = 11)			PE before 37 + 0 weeks (*n* = 30)			No PE before 37 + 0 weeks (*n* = 2611)		
	Biochemical markers measured at:		*P*	Biochemical markers measured at:		*P*	Biochemical markers measured at:		*P*
	8 + 0 to 10 + 6 weeks (*n* = 5)	11 + 0 to 13 + 6 weeks (*n* = 6)		8 + 0 to 10 + 6 weeks (*n* = 16)	11 + 0 to 13 + 6 weeks (*n* = 14)		8 + 0 to 10 + 6 weeks (*n* = 1659)	11 + 0 to 13 + 6 weeks (*n* = 952)	
Age (years)	34.0 (34.0–37.0)	34.5 (31.3–37.0)	0.642	34.0 (28.8–37.0)	37.0 (32.8–38.0)	0.143	32.0 (28.0–36.0)	32.0 (28.0–36.0)	0.222
BMI (kg/m^2^)	23.2 (22.7–32.1)	23.1 (22.2–25.5)	0.537	25.1 (23.2–28.7)	23.1 (21.8–26.0)	0.096	23.9 (21.4–27.4)	23.8 (21.2–27.5)	0.521
Ethnicity			0.455			0.734			< 0.001
White	4 (80.0)	6 (100)		13 (81.3)	12 (85.7)		1441 (86.9)	768 (80.7)	< 0.001
Black	0 (0)	0 (0)		0 (0)	1 (7.1)		34 (2.0)	37 (3.9)	0.008
Mixed	1 (20.0)	0 (0)		2 (12.5)	0 (0)		109 (6.6)	100 (10.5)	< 0.001
Asian	0 (0)	0 (0)		1 (6.3)	1 (7.1)		40 (2.4)	23 (2.4)	1.0
South‐East Asian	0 (0)	0 (0)		0 (0)	0 (0)		35 (2.1)	24 (2.5)	0.497
Smoker	1 (20.0)	0 (0)	0.455	1 (6.3)	2 (14.3)	0.586	214 (12.9)	95 (10.0)	0.028
ART	0 (0)	1 (16.7)	1.0	0 (0)	2 (14.3)	0.209	60 (3.6)	33 (3.5)	0.731
Insemination	0 (0)	1 (16.7)		0 (0)	1 (7.1)		10 (0.6)	6 (0.6)	
IVF	0 (0)	0 (0)		0 (0)	0 (0)		31 (1.9)	21 (2.2)	
IVF with egg donation	0 (0)	0 (0)		0 (0)	1 (7.1)		19 (1.1)	6 (0.6)	
Medical history			1.0			1.0			0.695
Chronic hypertension	2 (40.0)	1 (16.7)		3 (18.8)	2 (14.3)		15 (0.9)	9 (0.9)	
Diabetes mellitus	0 (0)	0 (0)		1 (6.3)	0 (0)		25 (1.5)	10 (1.1)	
Autoimmune disease	0 (0)	0 (0)		1 (6.3)	2 (14.3)		63 (3.8)	42 (4.4)	
APS	0 (0)	0 (0)		1 (6.3)	0 (0)		4 (0.2)	4 (0.4)	
Obstetric history			0.250			0.484			0.002
Nulliparous	0 (0)	2 (33.3)		6 (37.5)	7 (50.0)		813 (49.0)	406 (42.6)	0.002
Previous PE	1 (20.0)	1 (16.7)		4 (25.0)	1 (7.1)		16 (1.0)	14 (1.5)	0.256
Previous FGR	0 (0)	0 (0)		0 (0)	1 (7.1)		18 (1.1)	5 (0.5)	0.191
Biophysical variable									
GA at first‐trimester US (weeks)	12.6 (12.1–12.6)	13.1 (12.6–13.2)	0.116	12.7 (12.1–12.9)	12.6 (12.3–13.3)	0.530	12.6 (12.1–12.9)	12.9 (12.4–13.3)	< 0.001
MAP (mmHg)	94.3 (91.0–101.7)	96.3 (89.5–104.9)	0.931	90.5 (87.9–93.8)	94.5 (83.0–104.1)	0.693	84.0 (78.7–90.3)	84.7 (78.3–90.7)	0.829
MAP MoM	1.14 (1.10–1.37)	1.22 (1.14–1.22)	0.931	1.14 (1.10–1.21)	1.16 (1.05–1.30)	0.866	1.05 (0.97–1.14)	1.06 (0.97–1.14)	0.844
Mean UtA‐PI	2.54 (2.25–2.80)	1.89 (1.88–2.76)	0.247	2.15 (1.71–2.32)	1.89 (1.71–2.21)	0.618	1.69 (1.36–2.08)	1.65 (1.31–2.00)	0.002
Mean UtA‐PI MoM	1.67 (1.19–1.81)	1.18 (1.12–1.91)	0.537	1.24 (1.06–1.46)	1.12 (1.00–1.33)	0.552	1.03 (0.84–1.26)	1.03 (0.83–1.26)	0.834
Biochemical variable									
GA at PAPP‐A + PlGF measurement (weeks)	9.9 (9.6–10.3)	12.1 (11.5–12.3)	0.008	10.0 (9.5–10.5)	11.8 (11.4–12.2)	< 0.001	10.1 (9.7–10.6)	11.6 (11.1–12.3)	< 0.001
PAPP‐A (mU/L)	607.3 (602.3–1119.0)	1801.0 (1447.0–2201.5)	0.177	604.8 (307.8–1071.0)	1869.5 (1387.0–3513.5)	< 0.001	1033.0 (655.9–1575.5)	2395.5 (1499.0–3832.3)	< 0.001
PAPP‐A MoM	0.93 (0.71–1.09)	0.69 (0.61–0.74)	0.314	0.68 (0.53–1.06)	0.73 (0.61–0.85)	0.574	1.05 (0.74–1.50)	1.06 (0.72–1.51)	0.769
PlGF (pg/mL)	19.77 (19.03–22.35)	27.00 (20.69–40.15)	0.329	22.67 (19.00–26.24)	30.60 (25.10–47.48)	0.017	28.23 (22.0–35.89)	41.36 (31.92–54.40)	< 0.001
PlGF MoM	0.65 (0.62–0.86)	0.71 (0.45–0.97)	1.0	0.85 (0.70–0.96)	0.69 (0.52–1.00)	0.257	0.96 (0.77–1.19)	0.95 (0.74–1.19)	0.256

Data are reported as median (interquartile range) or *n* (%).

APS, antiphospholipid syndrome; ART, assisted reproductive technology; BMI, body mass index; FGR, fetal growth restriction; GA, gestational age; IVF, *in‐vitro* fertilization; MAP, mean arterial pressure; MoM, multiples of the median; UtA‐PI, uterine artery pulsatility index; US, ultrasound.

In the prediction of early‐onset PE and preterm PE using the Gaussian algorithm, no significant differences were observed in the areas under the ROC curves (AUCs) for any of the combinations of markers evaluated when the biochemical markers were assessed at 8 + 0 to 10 + 6 weeks compared with 11 + 0 to 13 + 6 weeks. Additionally, no substantial differences were observed in the detection rates at fixed 5% and 10% FPRs. The predictive capacity and detection rates in screening for early‐onset and preterm PE by the markers involved in the Gaussian algorithm are shown in Tables 2 and 3, respectively.

**Table 2:** Detection rate (DR) and area under receiver‐operating‐characteristics curve (AUC) for prediction of pre‐eclampsia before 34 weeks' gestation by Gaussian model, according to whether pregnancy‐associated plasma protein‐A (PAPP‐A) and placental growth factor (PlGF) were measured before or after 11 weeks

Method of screening	8 + 0 to 10 + 6 weeks (*n* = 5)			11 + 0 to 13 + 6 weeks (*n* = 6)			*P* *
	AUC (95% CI)	DR (% (95% CI)) at:		AUC (95% CI)	DR (% (95% CI)) at:		
		5% FPR	10% FPR		5% FPR	10% FPR	
*A‐priori* risk plus:							
MAP	0.746 (0.540–0.952)	40.0 (0–80.0)	40.0 (0–80.0)	0.887 (0.818–0.956)	33.3 (0–66.7)	50.0 (0–83.3)	0.364
UtA‐PI	0.863 (0.700–1.000)	60.0 (20.0–100)	60.0 (20.0–100)	0.812 (0.649–0.974)	33.3 (0–66.7)	50.0 (16.7–83.3)	0.733
PAPP‐A	0.615 (0.279–0.951)	20.0 (0–60.0)	40.0 (0–80.0)	0.803 (0.626–0.980)	33.3 (0–66.7)	50.0 (0–83.3)	0.277
PlGF	0.829 (0.732–0.927)	40.0 (0–80.0)	40.0 (0–80.0)	0.712 (0.479–0.945)	33.3 (0–66.7)	33.3 (0–66.7)	0.478
PlGF + UtA‐PI	0.923 (0.844–1.000)	60.0 (20.0–100)	60.0 (20.0–100)	0.841 (0.687–0.995)	50.0 (16.7–83.3)	50.0 (16.7–83.3)	0.530
MAP + PlGF	0.864 (0.746–0.982)	40.0 (0–80.0)	40.0 (0–80.0)	0.936 (0.894–0.978)	33.3 (0–66.7)	66.7 (33.3–100)	0.566
MAP + UtA‐PI	0.901 (0.793–1.000)	60.0 (20.0–100)	60.0 (20.0–100)	0.916 (0.822–1.000)	66.7 (33.3–100)	66.7 (33.3–100)	0.901
PAPP‐A + PlGF	0.790 (0.684–0.896)	20.0 (0–60.0)	40.0 (0–80.0)	0.751 (0.556–0.946)	33.3 (0–66.7)	50.0 (16.7–83.3)	0.816
MAP + PAPP‐A	0.744 (0.542–0.947)	40.0 (0–80.0)	40.0 (0–80.0)	0.910 (0.853–0.968)	33.3 (0–66.7)	66.7 (16.7–100)	0.272
UtA‐PI + PAPP‐A	0.849 (0.666–1.000)	60.0 (20.0–100)	60.0 (20.0–100)	0.846 (0.707–0.986)	50.0 (16.7–83.3)	50.0 (16.7–83.3)	0.984
MAP + UtA‐PI + PAPP‐A	0.891 (0.772–1.000)	60.0 (20.0–100)	60.0 (20.0–100)	0.929 (0.850–1.000)	66.7 (33.3–100)	66.7 (33.3–100)	0.752
MAP + UtA‐PI + PlGF	0.943 (0.881–1.000)	60.0 (20.0–100)	80.0 (20.0–100)	0.958 (0.925–0.990)	50.0 (16.7–83.3)	83.3 (50.0–100)	0.871
MAP + PAPP‐A + PlGF	0.838 (0.709–0.967)	40.0 (0–80.0)	40.0 (0–80.0)	0.930 (0.889–0.971)	33.3 (0–73.9)	66.7 (16.7–100)	0.488
UtA‐PI + PlGF + PAPP‐A	0.912 (0.820–1.000)	60.0 (20.0–100)	60.0 (20.0–100)	0.842 (0.689–0.995)	50.0 (16.7–83.3)	50.0 (16.7–83.3)	0.600
MAP + UtA‐PI + PlGF + PAPP‐A	0.935 (0.866–1.000)	60.0 (20.0–100)	80.0 (20.0–100)	0.950 (0.915–0.986)	50.0 (16.7–83.3)	83.3 (50.0–100)	0.879

* Comparison between AUCs was by two‐tailed *P*‐value.

FPR, false‐positive rate; MAP, mean arterial pressure; UtA‐PI, uterine artery pulsatility index.

**Table 3:** Detection rate (DR) and area under receiver‐operating‐characteristics curve (AUC) for prediction of pre‐eclampsia before 37 weeks' gestation by Gaussian model, according to whether pregnancy‐associated plasma protein‐A (PAPP‐A) and placental growth factor (PlGF) were measured before or after 11 weeks

	8 + 0 to 10 + 6 weeks (*n* = 16)			11 + 0 to 13 + 6 weeks (*n* = 14)			
		DR (% (95% CI)) at:			DR (% (95% CI)) at:		
Method of screening	AUC (95% CI)	5% FPR	10% FPR	AUC (95% CI)	5% FPR	10% FPR	*P* *
*A‐priori* risk plus:							
MAP	0.757 (0.647–0.866)	25.0 (6.3–43.8)	37.5 (18.8–68.8)	0.736 (0.590–0.883)	28.6 (7.1–50.0)	35.7 (7.1–64.3)	0.841
UtA‐PI	0.724 (0.564–0.884)	43.8 (18.8–68.8)	50.0 (25.0–75.0)	0.688 (0.541–0.835)	28.6 (7.1–50.0)	35.7 (14.3–64.3)	0.738
PAPP‐A	0.678 (0.520–0.835)	25.0 (6.3–50.0)	43.8 (18.8–68.8)	0.748 (0.621–0.875)	14.3 (0–35.7)	35.7 (7.1–64.3)	0.511
PlGF	0.719 (0.593–0.846)	25.0 (6.3–43.8)	25.0 (6.3–43.8)	0.712 (0.569–0.855)	35.7 (14.3–64.3)	35.7 (14.3–64.3)	0.948
PlGF + UtA‐PI	0.746 (0.589–0.903)	37.5 (12.5–62.5)	43.8 (18.8–68.8)	0.719 (0.571–0.866)	42.9 (14.3–71.4)	42.9 (14.3–71.4)	0.798
MAP + PlGF	0.802 (0.717–0.888)	25.0 (6.3–50.0)	31.3 (12.5–56.3)	0.799 (0.654–0.944)	28.6 (7.1–57.1)	57.1 (28.6–85.7)	0.976
MAP + UtA‐PI	0.798 (0.701–0.894)	31.3 (12.5–56.3)	31.3 (12.5–56.3)	0.765 (0.604–0.926)	42.9 (14.3–71.4)	50.0 (21.4–78.6)	0.743
PAPP‐A + PlGF	0.723 (0.595–0.851)	25.0 (6.3–43.8)	25.0 (6.3–50.0)	0.777 (0.648–0.906)	35.7 (14.3–64.3)	42.9 (21.4–71.4)	0.636
MAP + PAPP‐A	0.780 (0.678–0.883)	37.5 (18.8–62.5)	43.8 (18.8–68.8)	0.765 (0.620–0.910)	28.6 (7.1–50.0)	42.9 (14.3–71.4)	0.883
UtA‐PI + PAPP‐A	0.744 (0.585–0.902)	43.8 (18.8–68.8)	50.0 (25.0–75.0)	0.709 (0.565–0.853)	21.4 (7.1–50.0)	35.7 (14.3–64.3)	0.742
MAP + UtA‐PI + PAPP‐A	0.815 (0.720–0.909)	31.3 (12.5–56.3)	37.5 (12.5–62.5)	0.779 (0.619–0.938)	42.9 (21.4–71.4)	50.0 (28.4–78.6)	0.714
MAP + UtA‐PI + PlGF	0.804 (0.687–0.920)	31.3 (12.5–56.3)	37.5 (12.5–62.5)	0.793 (0.636–0.950)	42.9 (21.4–71.4)	50.0 (21.4–78.6)	0.911
MAP + PAPP‐A + PlGF	0.803 (0.721–0.885)	31.3 (12.5–62.5)	37.5 (12.5–62.5)	0.801 (0.660–0.941)	28.6 (7.1–57.1)	50.0 (21.4–78.6)	0.984
UtA‐PI + PlGF + PAPP‐A	0.749 (0.592–0.906)	37.5 (12.5–62.5)	43.8 (18.8–68.8)	0.722 (0.575–0.969)	42.9 (14.3–71.4)	42.9 (14.3–71.4)	0.798
MAP + UtA‐PI + PlGF + PAPP‐A	0.772 (0.639–0.904)	31.3 (12.5–56.3)	50.0 (25.0–75.0)	0.795 (0.640–0.950)	35.7 (14.3–64.3)	64.3 (35.7–85.7)	0.803

* Comparison between AUCs was by two‐tailed *P*‐value.

FPR, false‐positive rate; MAP, mean arterial pressure; UtA‐PI, uterine artery pulsatility index.

In the prediction of early‐onset PE using the FMF algorithm, no significant differences were observed in the AUCs for any of the combinations of markers evaluated when the biochemical markers were assessed before *vs* after 11 weeks, except for the combination of PAPP‐A and MAP, which showed a greater AUC when PAPP‐A was measured at or after 11 weeks. However, despite this significant difference, no substantial differences were observed in the detection rates at fixed 5% and 10% FPRs. The predictive capacity and detection rates in screening for early‐onset PE using the FMF algorithm are shown in Table 4.

**Table 4:** Detection rate (DR) and area under receiver‐operating‐characteristics curve (AUC) for prediction of pre‐eclampsia before 34 weeks' gestation by Fetal Medicine Foundation competing‐risks model, according to whether pregnancy‐associated plasma protein‐A (PAPP‐A) and placental growth factor (PlGF) were measured before or after 11 weeks

	8 + 0 to 10 + 6 weeks (*n* = 5)			11 + 0 to 13 + 6 weeks (*n* = 6)			
		DR (% (95% CI)) at:			DR (% (95% CI)) at:		
Method of screening	AUC (95% CI)	5% FPR	10% FPR	AUC (95% CI)	5% FPR	10% FPR	*P* *
*A‐priori* risk plus:							
MAP	0.655 (0.377–0.934)	40.0 (0–80.0)	40.0 (0–80.0)	0.844 (0.778–0.909)	16.7 (0–50.0)	16.7 (0–50.0)	0.259
UtA‐PI	0.744 (0.478–1.000)	40.0 (0–80.0)	40.0 (0–80.0)	0.809 (0.610–1.000)	33.3 (0–66.7)	66.7 (33.3–100)	0.697
PAPP‐A	0.472 (0.098–0.846)	40.0 (0–80.0)	40.0 (0–80.0)	0.750 (0.547–0.954)	33.3 (0–66.7)	33.3 (0–66.7)	0.105
PlGF	0.742 (0.540–0.944)	20.0 (0–60.0)	40.0 (0–80.0)	0.752 (0.509–0.995)	33.3 (0–66.7)	33.3 (0–66.7)	0.954
PlGF + UtA‐PI	0.856 (0.716–0.995)	40.0 (0–80.0)	60.0 (20.0–100)	0.838 (0.622–1.000)	50.0 (16.3–83.3)	66.7 (16.7–100)	0.903
MAP + PlGF	0.801 (0.630–0.972)	40.0 (0–80.0)	40.0 (0–80.0)	0.905 (0.833–0.977)	33.3 (0–83.3)	50.0 (16.7–83.3)	0.473
MAP + UtA‐PI	0.812 (0.623–1.000)	40.0 (0–80.0)	40.0 (0–80.0)	0.914 (0.838–0.990)	50.0 (16.7–83.3)	66.7 (16.7–100)	0.470
PAPP‐A + PlGF	0.723 (0.517–0.930)	20.0 (0–60.0)	40.0 (0–80.0)	0.769 (0.540–0.997)	33.3 (0–66.7)	33.3 (0–83.3)	0.790
MAP + PAPP‐A	0.356 (0.075–0.638)	40.0 (0–80.0)	40.0 (0–80.0)	0.892 (0.844–0.941)	16.7 (0–50.0)	50.0 (16.7–83.3)	< 0.001
UtA‐PI + PAPP‐A	0.719 (0.426–1.000)	40.0 (0–80.0)	60.0 (20.0–100)	0.855 (0.697–1.000)	50.0 (0–83.3)	66.7 (33.3–100)	0.403
MAP + UtA‐PI + PAPP‐A	0.790 (0.580–0.999)	40.0 (0–80.0)	40.0 (0–80.0)	0.937 (0.881–0.992)	50.0 (16.7–83.3)	66.7 (33.3–100)	0.292
MAP + UtA‐PI + PlGF	0.886 (0.770–1.000)	40.0 (0–80.0)	60.0 (20.0–100)	0.954 (0.905–1.000)	83.3 (33.3–100)	83.3 (50.0–100)	0.552
MAP + PAPP‐A + PlGF	0.789 (0.617–0.961)	40.0 (0–80.0)	40.0 (0–80.0)	0.916 (0.853–0.978)	50.0 (0–83.3)	50.0 (16.7–100)	0.379
UtA‐PI + PlGF + PAPP‐A	0.847 (0.700–0.994)	40.0 (0–80.0)	60.0 (20.0–100)	0.847 (0.642–1.000)	50.0 (16.7–83.3)	66.7 (16.7–100)	1.00
MAP + UtA‐PI + PlGF + PAPP‐A	0.926 (0.838–1.000)	60.0 (20.0–100)	80.0 (40.0–100)	0.967 (0.944–0.989)	66.7 (33.3–100)	100 (100–100)	0.669

* Comparison between AUCs was by two‐tailed *P*‐value.

FPR, false‐positive rate; MAP, mean arterial pressure; UtA‐PI, uterine artery pulsatility index.

In the prediction of preterm PE using the FMF algorithm, no significant differences were observed in the AUCs for any of the combinations of markers evaluated when PAPP‐A and PlGF were assessed before, compared with at or after, 11 weeks. In addition, no substantial differences were observed in the detection rates at fixed 5% and 10% FPRs. The predictive capacity and detection rates in screening for preterm PE using the FMF algorithm are shown in Table 5.

**Table 5:** Detection rate (DR) and area under receiver‐operating‐characteristics curve (AUC) for prediction of pre‐eclampsia before 37 weeks' gestation by Fetal Medicine Foundation competing‐risks model, according to whether pregnancy‐associated plasma protein‐A (PAPP‐A) and placental growth factor (PlGF) were measured before or after 11 weeks

	8 + 0 to 10 + 6 weeks (*n* = 16)			11 + 0 to 13 + 6 weeks (*n* = 14)			
		DR (% (95% CI)) at:			DR (% (95% CI)) at:		
Method of screening	AUC (95% CI)	5% FPR	10% FPR	AUC (95% CI)	5% FPR	10% FPR	*P* *
*A‐priori* risk plus:							
MAP	0.722 (0.604–0.840)	31.3 (12.5–56.3)	31.3 (12.5–56.3)	0.734 (0.593–0.875)	21.4 (0–42.9)	28.6 (7.1–50.0)	0.910
UtA‐PI	0.749 (0.617–0.882)	18.8 (0–37.7)	43.8 (25.0–68.8)	0.721 (0.566–0.876)	21.4 (0–42.9)	50.0 (28.4–78.6)	0.791
PAPP‐A	0.689 (0.529–0.850)	12.5 (0–37.5)	31.3 (12.5–56.3)	0.722 (0.592–0.853)	21.4 (0–42.9)	35.7 (14.3–57.1)	0.759
PlGF	0.738 (0.633–0.842)	12.5 (0–37.5)	31.3 (12.5–56.3)	0.737 (0.593–0.880)	35.7 (14.3–57.1)	35.7 (14.3–57.1)	0.992
PlGF + UtA‐PI	0.746 (0.637–0.855)	18.8 (0–43.8)	37.5 (18.8–62.5)	0.745 (0.603–0.886)	42.9 (21.4–71.4)	50.0 (21.4–78.6)	0.992
MAP + PlGF	0.785 (0.700–0.869)	31.3 (6.3–50.0)	37.5 (18.8–62.5)	0.797 (0.659–0.936)	35.7 (14.3–64.3)	42.9 (21.4–71.4)	0.904
MAP + UtA‐PI	0.803 (0.721–0.885)	25.0 (6.3–50.0)	37.5 (18.8–62.5)	0.766 (0.605–0.926)	35.7 (14.3–64.3)	50.0 (21.4–78.6)	0.712
PAPP‐A + PlGF	0.746 (0.637–0.855)	12.5 (0–43.8)	43.8 (18.8–68.8)	0.745 (0.603–0.886)	35.7 (14.3–64.3)	35.7 (14.3–64.3)	0.992
MAP + PAPP‐A	0.741 (0.625–0.857)	31.3 (6.3–56.3)	43.8 (18.8–68.8)	0.754 (0.612–0.896)	14.3 (0–42.9)	28.6 (7.1–57.1)	0.901
UtA‐PI + PAPP‐A	0.767 (0.627–0.907)	37.5 (12.5–68.8)	56.3 (31.3–81.3)	0.751 (0.610–0.892)	28.6 (7.1–57.1)	50.0 (29.9–78.6)	0.877
MAP + UtA‐PI + PAPP‐A	0.815 (0.726–0.905)	31.3 (12.5–56.3)	43.8 (18.8–68.8)	0.783 (0.624–0.942)	35.7 (14.3–57.1)	57.1 (28.6–85.7)	0.744
MAP + UtA‐PI + PlGF	0.830 (0.760–0.899)	18.8 (0–37.7)	43.8 (18.8–68.8)	0.804 (0.649–0.958)	42.9 (14.3–71.4)	57.1 (28.6–85.7)	0.785
MAP + PAPP‐A + PlGF	0.793 (0.709–0.877)	31.3 (12.5–56.3)	43.8 (18.8–68.8)	0.803 (0.665–0.942)	35.7 (14.3–64.3)	50.0 (21.4–78.6)	0.919
UtA‐PI + PlGF + PAPP‐A	0.789 (0.683–0.894)	25.0 (6.3–43.8)	37.5 (12.5–62.5)	0.764 (0.621–0.907)	42.9 (14.3–71.4)	50.0 (29.9–78.6)	0.805
MAP + UtA‐PI + PlGF + PAPP‐A	0.818 (0.713–0.924)	31.3 (6.3–50.0)	50.0 (25.0–81.3)	0.820 (0.669–0.971)	42.9 (14.3–71.4)	57.1 (28.6–85.7)	0.983

* Comparison between AUCs was by two‐tailed *P*‐value.

FPR, false‐positive rate; MAP, mean arterial pressure; UtA‐PI, uterine artery pulsatility index.

## DISCUSSION

This study provides evidence that multimarker algorithms have a similar performance in predicting early‐onset and preterm PE when PlGF and PAPP‐A are measured at 8 + 0 to 10 + 6 weeks or at 11 + 0 to 13 + 6 weeks' gestation. The timing of measurement of the serum biomarkers did not affect the performance of the Gaussian or the FMF algorithm in predicting preterm PE when PAPP‐A or PlGF was used alone or in combination with other markers. However, for the FMF model, the combination of PAPP‐A and MAP had a lower predictive capacity for early‐onset PE when PAPP‐A was measured before 11 weeks compared with at or after 11 weeks, which is probably due to the low number of cases with early‐onset PE.

A previous study evaluating the external validity of the available algorithms for the first‐trimester prediction of PE found that they underperformed if applied to an external population^15^. Additionally, PlGF seems to better identify patients at risk for PE when measured after 11 weeks^10^. Nonetheless, the performance of combined screening for early‐onset and preterm PE with biomarkers assessed at different points in gestation has not been assessed previously.

Our results have important clinical implications. They show that a two‐step approach to first‐trimester PE screening (combination of serum markers (PAPP‐A and PlGF) measured at 8–10 weeks and biophysical markers (MAP and UtA‐PI) measured at 11–13 weeks) is feasible, since its performance for predicting early‐onset and preterm PE does not deteriorate when serum biomarker levels are measured before 11 weeks. Additionally, a two‐step approach allows PE screening results to be provided immediately after the first‐trimester ultrasound assessment.

The main limitation of this study is the low number of cases with early‐onset PE and the relatively low number of cases with preterm PE; thus, our results should be considered with caution and validated in a larger cohort to ascertain whether the timing of biomarker assessment affects the screening performance of multimarker first‐trimester algorithms for the detection of early‐onset PE. Furthermore, there were statistically significant differences in baseline characteristics (ethnicity, smoking status, obstetric history, UtA‐PI and GA at ultrasound assessment) between the two cohorts of unaffected women, the most important being that the first‐trimester scan was performed slightly later in women in whom serum biomarkers were measured at or after 11 weeks (median GA, 12.9 *vs* 12.6 weeks). Nevertheless, this difference is small and probably not clinically significant. Finally, MAP was assessed by a single BP measurement in one arm, which has been shown to give significantly higher values than does assessment using the average of two measurements in both arms^16^. Since the latter is the methodology used in the FMF algorithm, it could have influenced its performance to predict PE in our cohort. However, the inaccuracies that might have resulted from the use of a single measurement for PE risk assessment by the FMF algorithm would have affected all participants similarly, therebyrendering the groups still comparable for the purpose of this study.

In conclusion, the Gaussian and the FMF algorithms have a similar performance in predicting early‐onset PE and preterm PE when PAPP‐A and PlGF are measured before or after 11 weeks, allowing the use of a two‐step risk assessment for PE. This approach allows immediate PE risk calculation at the time of the first‐trimester scan.
